# Effect of vitamin-D analogue on albuminuria in patients with non-dialysed chronic kidney disease stage 4–5: a retrospective single center study

**DOI:** 10.1186/1471-2369-13-102

**Published:** 2012-09-07

**Authors:** Hanne Skou Jørgensen, Simon Winther, Johan Vestergaard Povlsen, Per Ivarsen

**Affiliations:** 1 Department of Nephrology, Aarhus University Hospital, Aarhus N, DK-8200, Denmark

**Keywords:** Chronic kidney disease, Hyperparathyroidism, Vitamin D, Albuminuria, Alfacalcidol

## Abstract

**Background:**

The vitamin D receptor activator paricalcitol has been shown to reduce albuminuria. Whether this is a unique property of paricalcitol, or common to all vitamin D analogues, is unknown. The primary aim of this study was to evaluate the effect of alfacalcidol on proteinuria, measured as 24 hour (24 h) albuminuria, in patients with chronic kidney disease (CKD) stage 4–5 being treated for secondary hyperparathyroidism (sHPT).

**Methods:**

A retrospective single-center study including adult patients with CKD 4–5, undergoing treatment for sHPT with alfacalcidol, with macroalbuminuria in minimum one 24 h urine collection. Patients were identified in a prospectively collected database of all patients with S-creatinine > 300 μM or creatinine clearance < 30 ml/min. The observation period was from 1^st^ of January 2005 to 31^st^ of December 2009. Phosphate binders and alfacalcidol were provided to patients free of charge.

**Results:**

A total of 146 macroalbuminuric patients were identified, and of these, 59 started alfacalcidol treatment during the observation period. A 12% reduction in 24 h albuminuria was seen after starting treatment. In 19 patients with no change in renin-angiotensin-aldosteron-system (RAAS) inhibition, the reduction in albuminuria was 16%. The reduction remained stable over time (9%) in a subgroup of patients (n = 20) with several urine collections before and after the start of alfacalcidol-treatment.

**Conclusion:**

The present study supports experimental and clinical data on antiproteinuric actions of activated vitamin D analogues, and suggests that this may be a class-effect.

## Background

Proteinuria is a hallmark of kidney diseases, and a surrogate prognostic marker for progression of loss of kidney function in patients with chronic kidney disease (CKD)[1-3]. Reduction in proteinuria has been linked to improvement in both renal and cardiovascular outcomes [4,5]. Therefore, strategies to reduce proteinuria – inhibition of the renin-angiotensin-aldosteron-system (RAAS) with angiotensin converting enzyme inhibitors (ACEi) and angiotensin receptor blockers (ARBs) and blood pressure reduction - are currently the standard of care for CKD-patients [1]. Recently, activators of the vitamin D receptor (VDRAs) have been shown to reduce proteinuria.

VDRAs have been in use for treatment of the secondary hyperparathyroidism (sHPT) of CKD since the 1990s, but in the last decade, non-calcaemic actions of VDRAs have received much attention. Animal studies have demonstrated possible reno-protective effects of VDRAs, with reduction in proteinuria and glomerulosclerosis. The first clinical study to report an anti-proteinuric effect of VDRA was Agarwal et al. (2005) [6]. Using data from three randomized controlled trials (RCTs) comparing paricalcitol to placebo for treatment of sHPT, they reported a reduction in dipstick-proteinuria in 51% of treated patients compared to 25% in the placebo-group. The paricalcidol-dose was an average of 9.5 μg/week. Three RCTs have since confirmed this effect for paricalcitol [7-9], but whether the anti-proteinuric action is unique to this analogue, or might be common to all VDRAs, remains unknown. Recently one RCT reported a reduction in proteinuria when treating patients with IgA nephropathy with calcitriol [10], which indicates a class effect.

No studies have investigated the effect of alfalcalcidol on proteinuria**.** This pro-hormone (1(OH)D3) is widely used for treatment of sHPT in Europe, and it’s considerably more cost-effective than newer analogues. Two recent studies have investigated the efficacy and safety of alfacalcidol: A German observational study [11] of 1,159 CKD-patients found that alfacalcidol was efficient in controlling sHPT with few unwanted side-effects. In a randomized study comparing alfacalcidol and paricalcitol in the treatment of sHPT in haemodialysis patients, Hansen et al. (2011) [12] found, that the two analogues were equally effective and safe. Specifically, when titrated by treatment goals for PTH, calcium and phosphorus, they were equally effective in producing a 30% decrease in PTH with calcium and phosphorus remaining in the desired range.

We performed a single-centre retrospective study investigating the effects of alfacalcidol when used for treating sHPT in patients with CKD 4–5 and macroalbuminuria. Primary endpoint was urinary protein excretion, measured as 24-hour albuminuria. Secondary objectives were evaluation of possible effects on loss of kidney function (eGFR and creatinine clearance (CrCl)), blood pressure and s-calcium, s-phosphate and s-PTH.

## Methods

### Study population

Since 2004, all non-transplanted CKD 4–5 patients aiming for either dialysis or pre-emptive kidney transplantation have been prospectively registered in a database (PreReg®) at Aarhus University Hospital when their S-creatinine exceeded 300 μM or creatinine clearance declined below 30 ml/min. Data on underlying kidney diseases and albuminuria and kidney function based on a 24 h urine collection were included. After entering the PreReg programme, 24 h urine collections were typically repeated every 3–6 months until start of dialysis or transplantation. Data on s-PTH, s-calcium and s-phosphate, also measured every 3–6 months, were accessible from the electronic hospital register of biochemistry. Medications were registered in electronic patient records. Blood pressure and body weight were registered at all clinical visits, and these data were collected retrospectively from patient cards on the days when a 24 h urine was delivered.

In Denmark sHPT in CKD patients have been treated with activated vitamin D since the beginning of the 1990s. From 2005, treatment goals have been as described in K/DOQI guidelines, adapted to Danish conditions. According to local guidelines, alfacalcidol was used without cholecalciferol-supplementation. This study reflects daily clinical practice, where the individual physician decides when to start or change treatment. During the study period, both alfacalcidol and phosphate binders were provided to the patients free of charge.

From the beginning of 2005 to the end of 2009, 248 patients entered the database. Inclusion criterias for this study were: Adult patients aged >18 years, albuminuria > 300 mg/d in at least one 24 h urine collection and a minimum of two registered urine collections. By these, 146 patients were identified and included in the study. Last follow up was 31^st^ of December 2009.

### Parameters included in the analysis

*Demographics:* Sex, date of birth, diagnosis of kidney disease and entry date into the database.

*Blood-biochemistry:* S-creatinine, S-albumine, S-calcium, S-phosphate and S-PTH.

*24 h urine collection:* Volume, creatinine and albumin.

*Calculated values:* eGFR (4 point MDRD) and creatinine clearence (CrCl).

*Medications:* Vitamin D receptor activators, genuine vitamin D, phosphate binders, antihypertensives and diuretics.

*Blood pressure:* Systolic and diastolic.

### Ethics and legislation

This project was notified to, and approved by, the Danish Data Protection Agency, which governs the protection of personal data and the movements of such data in Denmark.

### Statistical

Mean ± SD and proportions were used to summarize the characteristics of the study samples. Continuous variables were compared by ANOVA repeated measurement, and ln transformation was performed when appropriate. Conventional student t-test and chi-square test were used as appropriate. STATA version 11.1 (Statagroup,Texas,USA) was used. *Time average albuminuria* used all 24 h urine collections in the dataset and weighted them for time before and after starting VDRA treatment.

## Results

### Baseline data

Baseline data by group are shown in Table 1. Of the 248 patients entering the database from 2005 to the end of 2009, 146 patients had albuminuria > 0.3 g/d at some point during follow up. Of these, 35 were already in treatment with alfacalcidol (aVDRA), 59 patients started treatment (VDRA+), and 52 did not receive a vitamin D receptor activator (VDRA-). Alfacalcidol was the only VDRA used.

**Table 1:** Baseline data by group

	**Already in VDRA treatment (aVDRA)**	**Starting VDRA treatment (VDRA+)**	**No VDRA treatment (VDRA-)**	**P**
N	35	59	52	
Age (mean, range)	59,4 (19–89)	57,2 (19–86)	65,1 (34–95)*	P < 0.05
Sex (% male)	71	71	63	Ns
**Diagnosis**				
Glomerulonephritis (n)	6	14	9	
Tubulointerstitiel nephritis (n)	1	1	1	
Pyeolonephritis (n)	1	0	1	
Obstructive nephropathy (n)	3	5	3	
Polycystic (ADPKD) (n)	0	5	4	
Hypertensive (n)	2	6	3	
DM type I (n)	0	7	4	
DM type II (n)	6	4	10	
Unknown (n)	13	11	13	
Other (n)	3	6	4	
**Observation time (days)**				
Total	17490	44282	30157	P < 0.05
Median (range)	500 (67–1603)	751(84–1716)	580 (93–1487)	P < 0.05
**Kidney Function**				
eGFR (ml/min/1.73 m2)	14.8 ± 5.8	16.5 ± 4.3	18.8 ± 10.9	Ns
Creatinine Clearance (ml/min)	19.2 ± 6.5	21.7 ± 15.6	23.8 ± 15.8	Ns
U-Albumin (g/24 h)	1.55 ± 1.96	1.96 ± 1.97	2.41 ± 2.32	Ns
**Mineral-Bone**				
S-calcium (mmol/l)	1.12 ± 0.11*	1.17 ± 0.07	1.21 ± 0.08	P < 0.001
S-phosphate (mmol/l)	1.67 ± 0.50*	1.45 ± 0.32	1.40 ± 0.32	P < 0.005
S-PTH (pmol/l)*	24.0 ± 16.0	21.3 ± 10.6	14.9 ± 10.1*	P < 0.005
**Blood pressure**				
Systolic (mmHg)	147 ± 19	151 ± 20	156 ± 20	Ns
Diastolic (mmHg)	85 ± 13	85 ± 11	82 ± 11	Ns
**Medication**				
Alfacalcidol (μg/week)	3.0 ± 1.7			
Calcium with Vitamin D (n,(%))	6 (17%)	4 (7%)	12 (23%)	Ns
Phosphate-binder (n,(%))	9 (25%)*	7 (12%)	7 (13%)	P < 0.01
ACEi/ARB (n,(%))	21(60%)1 dual	36 (61%)6 dual	24 (46%) 3 dual	Ns

Included are adult patients with at least one 24 h urine with albuminuria > 0.3 g/d. Mean ± SD if not otherwise stated. * This group differed significantly from the other two.

No significant difference was seen in eGFR or CrCl between groups. Untreated patients (VDRA- group) were significantly older, more frequent users of calcium-vitamin D tablets, and had lower s-PTH and s-phosphate with higher s-calcium compared to the VDRA + group.

During the observation period, 13 patients died, 4 were transferred to another center and 76 started dialysis or received a transplant. No difference was present between groups.

### Albuminuria before and after starting alfacalcidol (VDRA + −group)

Results for VDRA + group are shown in Table 2. There was a significant 12% (p < 0.05) decrease in albuminuria when comparing the last 24 h albuminuria before initiation of treatment, with the first collection after (VDRA + group, n = 59). Although presence of albuminuria was an inclusion criterion, two patients had no albuminuria immediately before starting alfacalcidol. They had macroalbuminuria at a later stage, and were thus included in the analyses to better reflect the variation in 24 h urine collections. The number of patients without albuminuria increased to nine after starting treatment (p = 0.06). In the untreated VDRA- group, 24 h albuminuria increased from 2.41 g/d to 2.69 g/d. The difference in change in albuminuria between the two groups (VDRA+/VDRA-) was significant (p < 0.05).

**Table 2:** Results in VDRA + −group

	**Before**	**After**	**P**
**Kidney Function**			
eGFR (ml/min/1,73 m2)	14.7 ± 4.3	13.0 ± 4.6	<0.001
U-Albumin (g/d)	1.81 ± 1.77	1.56 ± 1.57	<0.05
**Mineral-Bone**			
S-calcium (mmol/l)	1.15 ± 0.08	1.14 ± 0.07	ns
S-phosphate (mmol/l)	1.52 ± 0.35	1.56 ± 0.39	ns
S-PTH (pmol/l)	29.5 ± 14.9	26.0 ± 11.3	ns
**Blood Pressure**			
Systolic (mmHg)	151 ± 20	146 ± 19	<0.05
Diastolic (mmHg)	85 ± 11	83 ± 11	ns
**Antihypertensives: (n)**			
Angiotensin inhibitors*	21	22	
Angiotensin receptor blockers*	24	25	
Calcium antagonists	37	44	
Beta- blockers	28	35	
Others	5	9	

*5 dual in both groups.

Effects of starting treatment with alfacalcidol in 59 patients with secondary hyperparathyroidism and CKD 4–5. Mean ± SD.

Mean time between the two urine collections was 201 days. Alfacalcidol-dose was on average 2.4 μg/week (range 0.75 -7 μg/week) when the second 24 h urine was collected. eGFR decreased significantly during the observation period, from 14.7 to 13.0 ml/min, p > 0.001. The rate of loss of RRF was 5.8 ml/min/year. This was not statistically different from untreated patients (VDRA-), were the rate of loss of RRF was 7.1 ml/min/year (p = ns). No effect was seen on s-calcium, s-phosphate or s-PTH, despite the indication for treatment being sHPT. However, the number of patients taking phosphate binders increased from 9 to 20, indicating either loss of renal function or an effect of alfacalcidol treatment that was not reflected in a decrease in s-PTH. Systolic blood pressure decreased slightly (3%, p < 0.05), while diastolic pressure was unchanged. Overall, there were no marked changes in antihypertensive treatment.

In the VDRA + group, five patients did not receive ACEi/ARB-therapy. Of the remaining, 14 patients had no change while 25 patients increased and 15 patients decreased in dose. When analyzing patients with stable or no change (n = 19), the decrease in 24 h albuminuria was 16% (p < 0.005). Their eGFR decreased from 15.7 ± 1.3 ml/min to 13.8 ± 1.2 ml/min (ns). No significant changes were found in systolic or diastolic blood pressure, s-PTH, s-calcium and s-phosphate. The mean dose of alfacalcidol did not differ for this subgroup (2.46 μg/week).

### Longer observation period

**Figure 1:**
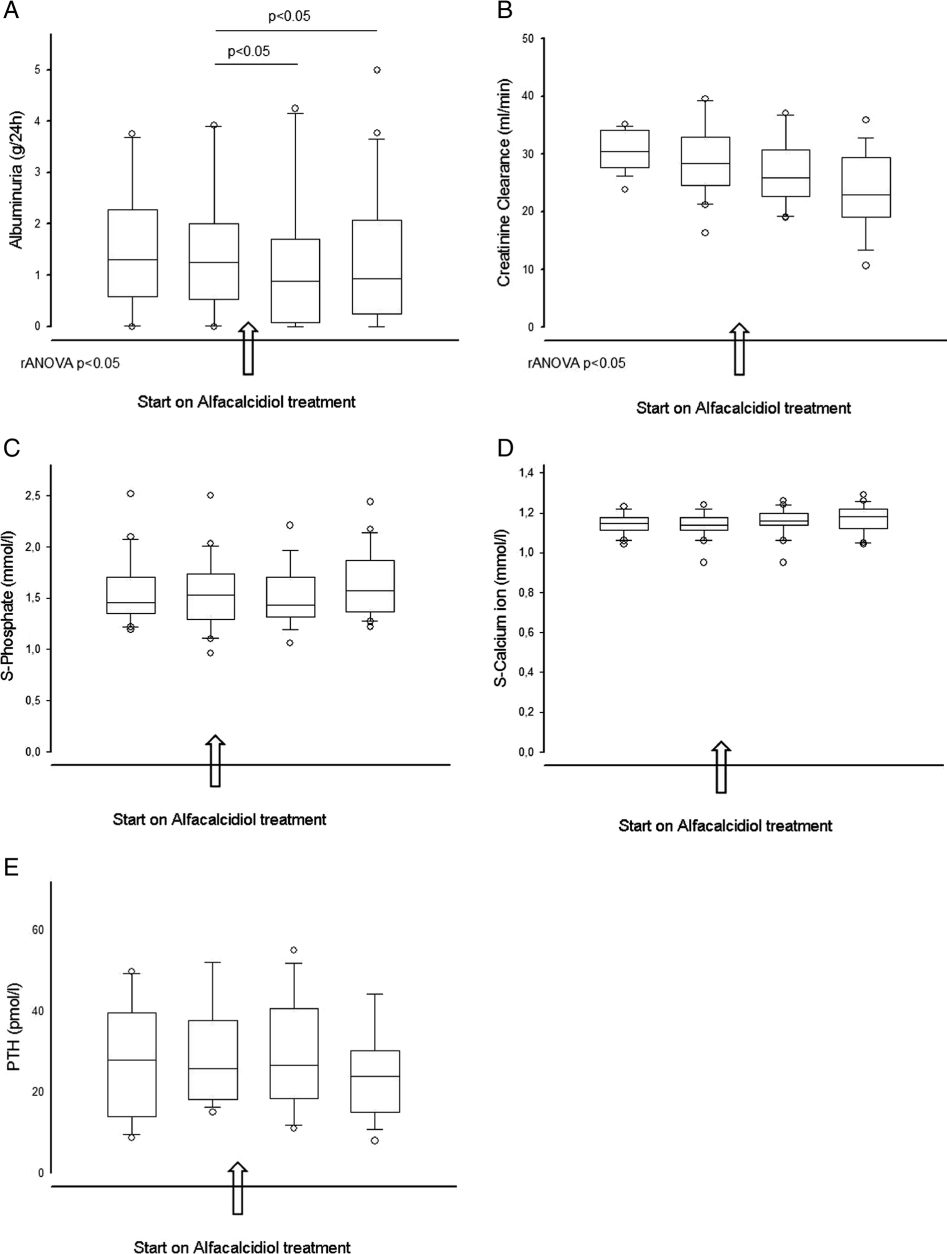
**Changes over time in 20 patients with two 24 h urinary collections both before and after starting treatment with alfacalcidol.** 1**A**: 24 h albuminuria, 1**B**: Creatinine clearance, 1**C**: S-phosphate, 1**D**: S-calcium and 1**E**: S-PTH. Data shown as median, Box 25 and 75% and whiskers 5 and 95%.

Results for this VDRA + subgroup are shown in Figure 1. Of the 59 patients who started alfacalcidol (VDRA+), 20 patients had at least two 24 h urine collections both before and after initiation of treatment. For this subgroup, the observation period was 324 days (median, range 210–819). Mean age was 57.8 years (range 30–83) with 65% males. Underlying renal diseases were: Chronic glomerulonephtis (7), unknown (5), diabetes (2), hypertension (2), polycystic kidney disease (2), tubulointerstitiel nephropathy (1) and obstructive nephropathy (1). Their average dose of alfacalcidol increased from 2.8 ± 1.6 μg/week to 3.5 ± 2.3 μg/week.

A significant and stable decrease (9%, p < 0.05) in 24 h albuminuria was demonstrated with time. Albuminuria was stable before initiation of VDRA, decreased significantly on alfacalcidol treatment and remained decreased during the observation period (Figure 1A). In comparison, a similar subgroup of 22 patients from the VDRA- population with minimum four 24 h urine collections, had a higher baseline value of albuminuria (2.73 ± 2.39 g/d), and showed no change during a period of 422 days (range 168–771) with albuminuria of 2.68 ± 2.19 g/d at the end of the observation period.

eGFR and CrCl decreased gradually over time (p < 0.001 and p > 0.005 respectively). The slopes did not change by starting alfacalcidol treatment (Figure 1B).

The number of patients treated with phosphate binders in the VDRA + subgroup increased from two at the time of the first urine collection, to eight at the end of the long observation period. No effect was seen on s-phosphate, s-calcium or s-PTH (Figures 1C, D, E).

Systolic and diastolic blood pressures were stable over time (data not given). Changes in antihypertensives were as follows: In ACEi/ARBs four patients had no change, five increased and six decreased doses, and the remaining five did not receive RAAS-blockade. Number of patients on calcium antagonists increased from 12 to 15, on betablocker from 10 to 12, on loop-diuretics from 13 to 15. The total dose of loop-diuretics increased from 1,905 mg/d to 2,445 mg/d. Three patients used thiazid diuretic during the study. Of these, two received an aldosterone-antagonist, this was reduced to one.

### Time-average albuminuria

Analysis of all data in the VDRA + group (n = 59) showed a small, but significant, decrease in albuminuria (8,6%, p = 0.05) after start of alfacalcidol treatment. eGFR decreased significantly (15%, p < 0.001) while the rate of decline of residual renal function (RRF) remained unchanged (Table 3). The rate of loss of RRF was comparable to, and not siginificantly different from, that of untreated patients (0.014 ml/min/d ± 0.025).

**Table 3:** Time weighted analysis in VDRA + −group

	**Before**	**After**	**P**
**Kidney Function**			
eGFR (ml/min)	14.8 ± 3.6	12.6 ± 5.5	<0.001
Delta eGFR (ml/min/day)	−0.018 ± 0.032	−0.013 ± 0.020	Ns
U-Albumin (g/d)	1.75 ± 1.64	1.60 ± 1.48	=0.05
**Mineral-Bone**			
S-calcium (mmol/l)	1.15 ± 0.06	1.15 ± 0.07	Ns
S-phosphate (mmol/l)	1.51 ± 0.28	1.62 ± 0.38	Ns
S-PTH (pmol/l)	26.7 ± 11.5	23.8 ± 11.2	Ns
**Blood Pressure**			
Systolic (mmHg)	147 ± 19	147 ± 17	Ns
Diastolic (mmHg)	82 ± 9	84 ± 10	Ns

Time average values of all 24 h urinary collections before and after starting treatment with alfacalcidol in 59 patients with CKD 4–5. Number of urinary collections varied from 1 to 4 before and from 1 to 6 after. The mean time follow up time was 422 days (range 66–1347) before start of alfacalcidol and 364 (72–1196) after. Mean ± SD.

## Discussion

In this retrospective study, treatment with alfacalcidol significantly reduced 24 hour albuminuria in a group of patients with CKD4-5, being treated for secondary hyperparathyroidism. The reduction was in the range of 8-12%, depending on the time frame analyzed. Patients with stable or no treatment with ACEi/ARBs had a greater decrease of 16%. The reduction in 24 hour albuminuria remained stable over time in an analysis of a subgroup of patients, where data were available for a longer observation period. No change was seen in the biochemical parameters of secondary hyperparathyroidism, although use of phosphate-binders did increase. Alfacalcidol-treatment did not significantly affect blood pressure, and loss of residual renal function was comparable to untreated patients.

Animal studies of VDRA-treatment in different experimental models of kidney failure support these clinical findings by consistently reporting reduction in albuminuria and also glomerulosclerosis [13-27]. Proposed mechanisms include effects on the RAAS-system, as demonstrated by VDR-knockout mice having increased renin expression and thereby increased angiotensin II-production [19,28]. Supportive of this, SNX-rats treated with VDRA show suppression of the biosynthesis of renin and angiotensin II [21]. When vitamin D-treatment is combined with ACEi/ARBs, additional reno-protection has been demonstrated [18,20,22,24,25]. Several other mechanisms may contribute: Reduced podocyte-damage with restoration of the glomerular filtration barrier [14,17,23,26], reduced inflammatory response with inhibition of neutrophile and monocyte cell-accumulation and reduction in chemoattractants[15,18], anti-proliferative effects[13-16,29] and reduced oxidative stress [27].

Our findings are in accordance with other clinical studies investigating the effects of VDRAs on proteinuria. Three randomized, placebo-controlled studies have used paricalcitol 1–2 μg/d as add-on-top treatment to stable RAAS-blockade with ACEi or ARBs. In a small RCT by Alborzi et al [7], 24 patients with CKD 3 were treated with 1 or 2 μg/d paricalcitol or placebo for 1 month. A 46-48% reduction in 24 hour albuminuria with either dose was reported, with an increase of 35% in the placebo-group. There was a concomitant decrease in inflammation, measured as hsCRP, which was greater with the higher dose of paricalcitol (20% reduction with 1 μg/d, and 30% reduction with 2 μg/d) compared to 50% increase with placebo. Fishbane et al [8] randomized 61 patients with CKD2-4 and proteinuria >400 mg/d to 1 μg/d paricalcitol or placebo. Half of these patients were diabetics and almost all in treatment with ACEi/ARBs (90.1%). With 1 μg/d of paricalcitol for 6 months there was a decrease in spot urine protein-creatinine ratio of 17.6% compared to an increase of 2.9% in controls. In the VITAL-study performed by de Zeeuw et al [9], 281 patients with type 2 diabetes, nephropathy and stable ACEi/ARB-therapy, were randomized to 1 or 2 μg/d paricalcitol or placebo for 24 weeks. Most had macroalbuminuria (72%). Results were reported primarily as urinary albumin-to-creatinine ratio (UACR), but 24 hour urinary albumin was also available for a large subgroup (82%). There was a reduction in UACR in both treated arms (14% and 20%) and also in 24 hour urinary albumin (10% and 34%), but only the substantially greater reduction in the 2 μg/d arm reached statistical significance.

The amount of urinary protein excretion at baseline differed substantially in these studies - it was approximately twice as high in the study-population of Fishbane et al. (proteinuria 2.6-2.7 g/d), compared to the diabetics of the VITAL-study (albuminuria 1.0-1.1 g/d). Higher excretion of protein may be related to a higher level of renal inflammation – and anti-inflammatory and immuno-modulatory actions of VDRAs might explain why Fishbane et al. found a significant, and quite high, reduction in proteinuria with a 1 μg/d dose, while the 1 μg/d arm in the VITAL-study did not reach statistical significance. Also, VDRAs may reduce *proteinuria*, as reported by Fishbane, more than *albuminuria*, as reported by the VITAL-group, as some of the urinary proteins measured are of tubular origin and VDRAs reduce interstitial inflammation.

In our study, the dose of alfacalcidol was modest – on average 3.0 +/− 1.7 μg/weekly, or less than 0.5 μg/daily. Even so, there was a significant effect on 24 hour albuminuria similar to the 1 μg arm of the VITAL-study. The amount of albuminuria was generally high, on average 2.0 g/d, and our study-population was heterogeneous in underlying kidney diseases, comparable to Fishbanes study-population. We also found a tendency towards a greater decline in albuminuria with higher alfacalcidol-dose, but this did not reach statistical significance.

The primary goal of treatment for our patients was sHPT, and not changes in urinary protein excretion. Alborzi et al [7] do not report baseline data of iPTH, but state, that they were unable to detect statistically significant reductions in PTH levels at 1 month. Fishbane et al [8] report slightly elevated iPTH at baseline (7.8 pmol/l) without difference between groups, with a significant and stable decrease over 6 months (5.1 pmol/l) with paricalcitol-treatment. In the VITAL-study [9], iPTH was higher at baseline (9.6-11 pmol/l), with a significant reduction of approximately 30% in both treated groups. Our patients differed from the abovementioned studies, in having a higher iPTH at baseline (26.7 pmol/L) and also more reduced kidney function, with mean eGFR < 20 ml/min.

Anti-proteinuric effects have been reported for the activated vitamin D hormone, calcitriol, as well. In an open-label study by Szeto et al [30], ten patients with IgA nephropathy and persistent proteinuria despite RAAS-blockade received calcitriol 0.5 μg/twice weekly, which produced a 27.8% decrease in spot urine protein-creatinine ratio over 12 weeks. Similar results were reported in a recent and larger RCT (n = 50) by Liu et al [10], where the same dose of calcitriol given over 48 weeks resulted in a 19% reduction in 24-hour urine protein excretion, with a between-group difference with placebo of 41%. Simultaneously, there was a decrease in serum TGF-β level.

### Strenghts and limitations

The retrospective design is a weakness of this study. The observation period was relatively short and most of the observations were based on patients with only one urine collection after starting treatment in addition to the baseline collection. The antihypertensive treatment and use of ACEi/ARBs and diuretics were not controlled, but followed daily practice in our out-patient clinic, representing a confounding factor. Dosage of alfacalcidol was modest, and although the indication was treatment of sHPT, no overall change in relevant biohemical parameters could be detected. This shows an inconsistency in following guidelines during day to day practice.

Decrease in residual renal function reflects loss of nephron mass and might as such accompany a decrease in albuminuria, but in the present study the VDRA- group had unchanged albuminuria despite a loss of kidney function in the same range as the VDRA + group. Our primary endpoint, albuminuria, is a surrogate marker for progression of loss of kidney function. Recently, a clinical trial of renin inhibitor aliskiren, (ALTITUDE-trial) [31] was closed, as it failed to demonstrate the anticipated beneficial effects on clinical endpoints (renal death, reaching end-stage renal disease and doubling of baseline serum creatinine clearance) despite achieving a greater reduction in proteinuria. It remains unknown whether VDRAs when used to reduce proteinuria might be beneficial in halting the progression of chronic kidney disease. Further prospective, randomized, placebo-controlled studies, investigating both changes in proteinuria, and clinically relevant renal endpoints are needed to clearify a possible role of VDRAs in the treatment of chronic kidney disease.

As alfacalcidol and phosphate binders were provided free of charge, the socio-economic status of patients as a bias was avoided.

To our knowledge, this is the first study to report the effects of alfacalcidol on proteinuria. This study supports experimental and clinical data that VDRAs have antiproteinuric actions, and suggests that this may be a class-effect.

## Competing interests

This study was supported by an unrestricted grant from Abbots Denmark. The results presented here have not been previously published in whole or in part.

## Authors’ contributions

All listed authors were involved in study design and acquisition of data, contributed to the analysis and interpretation of data and were involved in drafting the manuscript and revising it. The final version of this paper was approved by all authors.

## Disclosure

The study was supported by an unrestricted grant from Abbott Denmark.

## Pre-publication history

The pre-publication history for this paper can be accessed here:

http://www.biomedcentral.com/1471-2369/13/102/prepub
